# Mid-term power load forecasting using an ensemble deep learning model with BKA and CWGAN-GP enhancements

**DOI:** 10.1038/s41598-026-49674-x

**Published:** 2026-04-22

**Authors:** Shucheng Luo, Xiaohong Chen, Xinfu Pang, Baoshi Wang, Zedong Zheng

**Affiliations:** 1 Key Laboratory of Energy Saving and Controlling in Power System of Liaoning Province, Shenyang Institute of Engineering, Shenyang, 110136 People’s Republic of China; 2 School of Computing and Mathematical Sciences, University of Leicester, Leicester, LE1 7RH UK

**Keywords:** Mid-term load forecasting, Data augmentation, Hybrid deep learning, BKA optimization, XGBoost, Electrical and electronic engineering, Computational science

## Abstract

Electric load forecasting serves as a crucial decision-making foundation for the operation of power systems, offering important guidance for system scheduling optimization, cost reduction, and capacity planning. With the increasing penetration of new power electronic devices, such as electric vehicle charging stations, load volatility and nonlinearity have become more pronounced. At the same time, the scarcity of historical data for newly added loads often leads to challenges in achieving high forecasting accuracy. This paper proposes a hybrid deep learning model for mid-term electric load forecasting based on BKA optimization and CWGAN-GP data augmentation. First, the original load dataset is expanded through data augmentation using CWGAN-GP. Then, CNN-BiTransformer-BiLSTM and BiTCN-BiGRU-Attention models are independently trained on similar semi-monthly (14-day) datasets, producing two sets of validation prediction vectors. Subsequently, these vectors are combined with the validation set and used to train the final model via XGBoost. Finally, the features of the target semi-month are fed into the final model to obtain the forecasted results. Experimental results across three groups demonstrate that the proposed model reduces RMSE by at least 3.02% and lowers MAPE by at least 17.7%, validating its superior accuracy and robustness.

## Introduction

Electric load forecasting constitutes a critical basis for decision-making in the planning and operation of power systems^1^. Mid-term load forecasting typically refers to predicting electricity demand over a horizon ranging from one month to several years, covering a timescale between short-term and long-term forecasting. It primarily serves applications such as electricity market transactions, generation maintenance scheduling, and fuel procurement planning.

### Literature review

Mid-term load forecasting serves as a core underpinning for the safe, economical, and efficient operation of power systems. Its necessity arises from bridging the gap between long-term planning and short-term scheduling, directly impacting five critical domains: power generation, power grid, electricity market, end-users, and energy policies^2^. Moreover, it stands as a pivotal technology enabling the digital transformation of power systems amid the high penetration of renewable energy. Mid-term load forecasting models can generally be categorized into traditional white-box models based on statistical methods and data-driven black-box models. Statistical models, including linear regression, time series analysis, and exponential smoothing methods, explicitly describe the relationships between load and influencing factors through mathematical equations. In contrast, machine learning models, such as support vector machines, random forests, and artificial neural networks, offer powerful nonlinear fitting capabilities but are sensitive to data quality and often suffer from interpretability issues due to their “black-box” nature. With the widespread deployment of smart meters and Internet of Things technologies, deep learning models, represented by architectures such as LSTM and Transformer, have gained increasing attention for their strong capability to model temporal dependencies. As datasets become increasingly large and complex, data-driven approaches have emerged as the mainstream, and applying machine learning models for mid-term load forecasting has become a prevailing trend^3–7^.

Although data-driven machine learning models have been widely applied, a single model often struggles to adapt to the diverse scenarios encountered in power load forecasting^8–11^. Ensemble learning algorithms, by integrating the predictions of multiple heterogeneous base learners using parallel or sequential ensemble strategies^12^—such as meta-learner stacking in Stacking^13^, error correction and boosting in Boosting^14^, and soft/hard voting mechanisms in Voting^15^—construct composite models that leverage complementary strengths. These methods not only help mitigate overfitting risks through bias-variance decomposition theory but also exploit heterogeneous models’ diverse feature extraction capabilities.

Optimization algorithms refer to methods that, through specific strategies, enable the objective, namely the fitness function, to attain its optimal value, and they have been widely applied in hyperparameter selection for load forecasting models^16,17^. Swarm intelligence optimization algorithms, which simulate foraging and other behaviors observed in biological populations, have found extensive applications across various fields. Represented by algorithms such as PSO and GWO, swarm intelligence approaches construct distributed search mechanisms within a multidimensional solution space by mimicking the cooperative foraging behaviors of biological groups. In load forecasting, these algorithms are typically employed to optimize the hyperparameters of base prediction models, thereby enhancing the scientific rigor and reproducibility of model configurations.

Load data often exhibits considerable volatility and uncertainty in mid-term forecasting scenarios. Data augmentation can help reduce a model’s sensitivity to outliers and noisy data^18^. As an advanced data augmentation technique, Generative Adversarial Networks (GANs) have demonstrated remarkable success in numerous domains, including the power systems field, owing to their ability to model the underlying distribution of data^19–21^. The above important literature is summarized in Table 1.

**Table 1:** Recent relevant research review.

Reference	Decomposition algorithm	Optimization algorithm	Augmentation technique	Prediction model
Dostmohammadi M et al.^12^	None	Genetic Algorithm	Clustering analysis	Stacking of ANN, KNN, DT, RF, XGBoost, GBT
Ren X et al.^13^	CEEMDAN and Sample Entropy	Improved Arctic Puffin Optimization	K-fold cross-validation	Stacking(base learners and DDPG-based meta-learner)
Saini P et al.^14^	ISTD	None	None	PGBM(Gradient Boosting and Quantile Regression)
Khan SA et al.^15^	None	None	None	KNN, RF, DT, Voting Ensemble Regression
Wang Y et al. ^16^	None	IMPA(ODE and mutation)	KFCM clustering as input prep	SCN
Yamasaki M et al. ^17^	STL, EMD, EEMD, CEEMDAN and EWT	AutoML	None	GBR, XGBoost, kNN, SVR
Acción Á et al. ^18^	None	None	Dual-window Superpixel	Superpixel-based CNN
Bendaoud NMM et al. ^19^	None	None	None	CGAN
Bu X et al. ^20^	VMD	None	CGAN	Semi-supervised Regression
Hu Y et al. ^21^	None	None	Iterative data augmentation and GAN generator	Deep learning GAN framework

## Research gap and contributions

Although various deep learning approaches have been applied to mid-term load forecasting, several critical limitations still remain:

1. Most existing methods overlook the frequency characteristics of load sequences, treating the signal as an undivided whole, thereby failing to leverage its intrinsic modal structures and frequency-domain information.
2. Limited attention has been paid to data augmentation, which is crucial for enhancing model generalization, especially under conditions of limited or noisy historical data.
3. Many hybrid forecasting models adopt fixed architectures or rely on manually tuned hyperparameters, potentially constraining the adaptability and optimization potential of the models across different datasets or forecasting horizons.

In response to the above limitations, and considering the limited adaptability and challenges in modeling nonlinear relationships inherent in classical mid-term load forecasting methods^13^, this study proposes a Blending ensemble mid-term load forecasting model based on BKA optimization and CWGAN-GP data augmentation. The model integrates CNN-BiTransformer-BiLSTM and BiTCN-BiGRU-Attention architectures. The main contributions of this work are as follows:

1. Application of the FMD algorithm to mid-term load forecasting. To address the limitation of overlooking load sequence frequency characteristics, a novel model, CNN-BiTransformer-BiLSTM, is proposed and applied to mid-term load forecasting tasks.
2. Utilization of the BiTCN-BiGRU-Attention model for mid-term load forecasting. To compensate for the deficiency of fixed hybrid model architectures, a Blending ensemble model is introduced, in which CNN-BiTransformer-BiLSTM and BiTCN-BiGRU-Attention serve as base learners. Through the Blending integration strategy, the model leverages the strengths of different architectures, thereby improving forecasting accuracy and enhancing adaptability to diverse load variation patterns.
3. Application of CWGAN-GP-based data augmentation for mid-term load forecasting. To fill the research gap of insufficient attention to data augmentation, CWGAN-GP is employed to generate synthetic samples that conform to the real data distribution, thus enriching the diversity of the training dataset and improving the models’ ability to learn complex load fluctuations and enhance predictive performance.
4. Introduction of BKA for hyperparameter optimization. To solve the limitation of manual hyperparameter tuning, BKA is utilized to optimize hyperparameters for the FMD, CNN-BiTransformer-BiLSTM, and BiTCN-BiGRU-Attention models.

The structure of this paper is as follows: Section “Introduction” presents the introduction, outlining the significance of mid-term load forecasting, research progress, and the primary contributions of this study; Section “Methodology” elaborates on the full methodology of the model, including the overall forecasting framework, data preprocessing, and core model principles; Section “Simulation Experiments and Analysis” details the simulations and result analyses, including experimental conditions, dataset sources, comparative experiments, ablation studies, and visualization results; Section “Discussion” discusses the intrinsic reasons for the performance advantages of the proposed model, as well as its limitations in practical engineering applications; Section “Conclusion” summarizes the conclusions of this study and puts forward feasible prospects for future research directions in mid-term load forecasting.

## Methodology

**Fig. 1:**
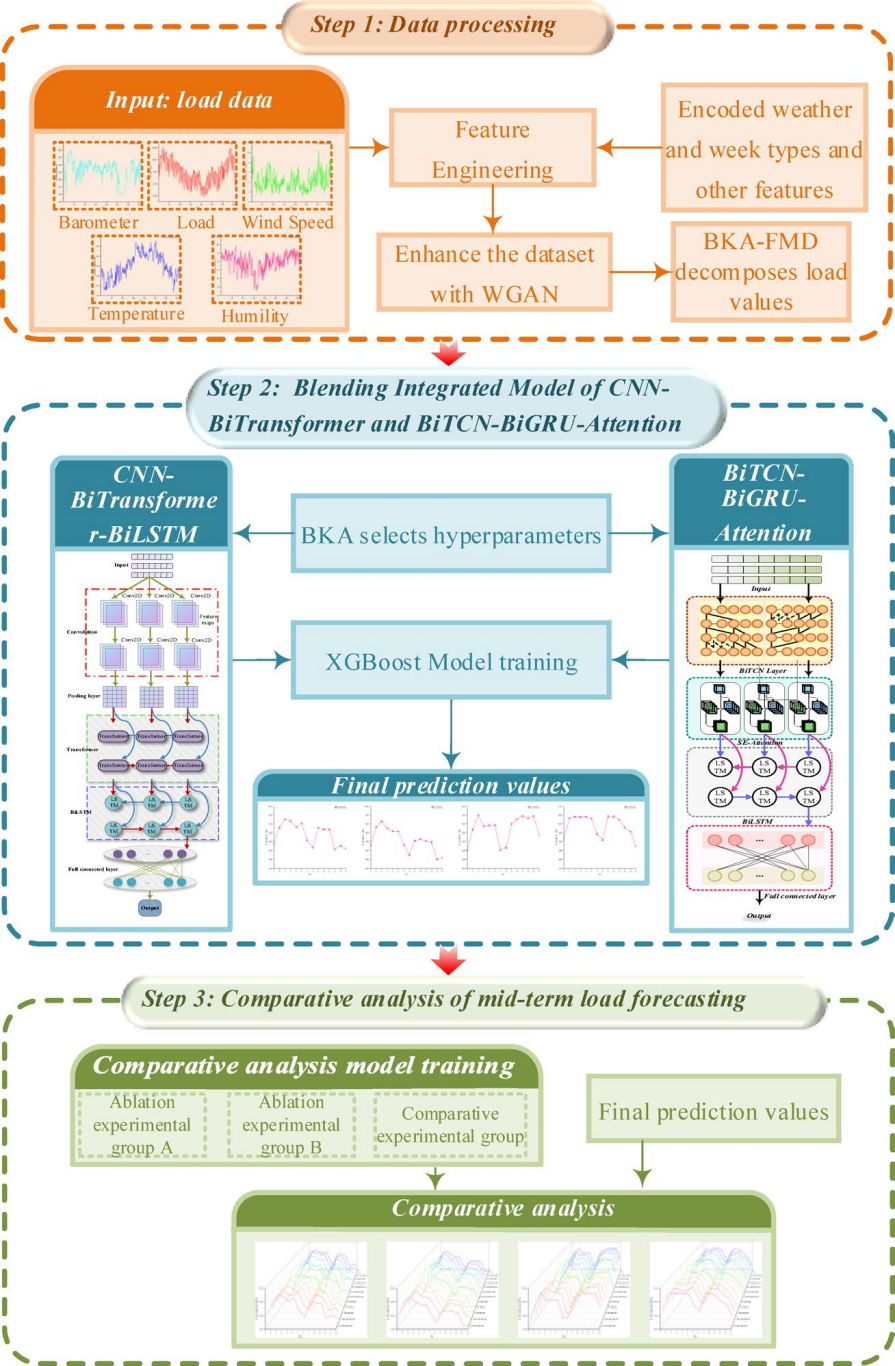
Structural diagram of the proposed algorithm’s forecasting and evaluation process.

The overall framework of the proposed approach is illustrated as follows, encompassing data preprocessing, model training, forecasting, and comparative analysis of prediction results:

Figure 1 illustrates the experimental procedure of this study:

*Step* 1: Data preprocessing. Periodic features such as date and time are encoded using periodic encoding. Through correlation analysis and factor analysis, features unrelated to load are removed, and new uncorrelated features are extracted, so as to optimize input quality and reduce model learning complexity. A synthetic dataset is generated using CWGAN-GP and combined with the original dataset to form an augmented training set, so as to enhance data diversity and generalization by generating realistic samples under conditions of limited or noisy data. Then, the load data is decomposed using FMD optimized by the BKA algorithm, and the resulting components are categorized into high-frequency and low-frequency components.

*Step* 2: Load forecasting. The low-frequency components are input into the CNN-BiTransformer-BiLSTM model, while the high-frequency components are fed into the BiTCN-BiGRU-Attention model, as these two base learners exhibit complementary adaptability to different frequency components. After training on the training set, both models generate prediction vectors on the validation set. These prediction vectors are treated as new features and appended to the validation set to construct an enhanced validation dataset. The XGBoost algorithm combines predictions from the base models to improve overall forecasting performance, and is then used to train this dataset and subsequently predict the half-month load for the test set.

*Step* 3: Comparative experiments. The prediction results are evaluated through two ablation studies and comparative analyses with several classical and state-of-the-art algorithms, verifying the proposed approach’s effectiveness, accuracy, and robustness.

### Data preprocessing

The accuracy of mid-term load forecasting is influenced by factors such as the distribution of outliers, scale differences, feature selection, and the sufficiency of the dataset^22,23^. To address these challenges, a series of data preprocessing steps is necessary. This study’s preprocessing includes outlier detection and handling, normalization, correlation analysis, factor analysis, data augmentation, and target decomposition. Outliers are identified by checking whether the load variation between adjacent time.

points exceeds 50%, and are handled by applying a moving average method for substitution. Normalization is performed using the min–max scaling approach. As these two techniques are commonly used, detailed explanations are omitted here.

#### Correlation analysis

To reduce interference with model convergence and lower computational cost, irrelevant variables should be filtered through correlation analysis. The elimination of unrelated variables based on correlation coefficients should consider both linear and monotonic relationships, namely, by computing the Pearson correlation coefficient and the Spearman rank correlation coefficient, and selecting the one with the highest significance. Before conducting a correlation analysis between each feature and the load, a K–S test^24^ should be applied to both features and the load to determine whether they follow a normal distribution. Features that do not follow a normal distribution are not subjected to Pearson correlation analysis. Finally, features not significantly correlated with the load under both tests, defined as having an absolute correlation coefficient less than 0.1, are removed.

1. Pearson correlation analysis.

The Pearson correlation coefficient is used to measure the strength of the linear relationship between two continuous variables:

1 $$r = \frac{{\mathop \sum \nolimits_{i = 1}^{n} \left( {X_{i} - \overline{X} } \right)\left( {Y_{i} - \overline{Y} } \right)}}{{\sqrt {\mathop \sum \nolimits_{i = 1}^{n} \left( {X_{i} - \overline{X} } \right)^{2} } \cdot \sqrt {\mathop \sum \nolimits_{i = 1}^{n} \left( {Y_{i} - \overline{Y} } \right)^{2} } }}$$

where $X_{i}$ and $Y_{i}$ are the *i*-th observations of variables $X$ and $Y$, respectively; $\overline{X}$ and $\overline{Y}$ are the sample means of variables $X$ and $Y$; and $n$ is the sample size. This coefficient ranges from -1 to 1, where 1 indicates a perfect positive linear relationship, -1 indicates a perfect negative linear relationship, and 0 indicates no linear relationship.

(2) Spearman rank correlation analysis.

The Spearman rank correlation coefficient is a non-parametric statistic used to assess the monotonic relationship between the ranks of two variables:

2 $$\rho = 1 - \frac{{6\mathop \sum \nolimits_{i = 1}^{n} d_{i}^{2} }}{{n\left( {n^{2} - 1} \right)}}$$

where $d_{i}$ is the difference in ranks of the *i*-th observation between the two variables, i.e.,$d_{i} = R\left( {X_{i} } \right) - R\left( {Y_{i} } \right)$; $R\left( {X_{i} } \right)$ and $R\left( {Y_{i} } \right)$ represent the ranks of the *i*-th observation in variables $X$ and $Y$, respectively; and $n$ is the sample size. The value of this coefficient also ranges from − 1 to 1, with the interpretation of the correlation being similar to that of the Pearson correlation coefficient.

#### Factor analysis

Features are often correlated, which means that multicollinearity may exist, leading to issues such as overfitting and increased computational cost^25^. Therefore, this study employs factor analysis to express the original features using fewer factor features, while ensuring that these factor features are as uncorrelated as possible. Principal component analysis (PCA) is used in this study to extract features.

The general formula for factor analysis is:

3 $$X = {\Lambda }F + \varepsilon$$

where $X$ is the vector of observed variables; ${\Lambda }$ is the factor loading matrix, representing the influence of each factor on the observed variables; $F$ is the vector of latent factors, representing unobserved variables; and $\varepsilon$ is the specific factor or error term, representing the portion of the observed variables that the factors cannot explain.

Covariance:

4 $$Cov\left( X \right) = \Lambda \Lambda^{\prime } + \psi$$

where ${\Psi }$ is the covariance matrix of the specific factors, typically a diagonal matrix representing the unique variance of each observed variable. The covariance value is used to measure the fluctuation information of the original features retained by the latent factors, or in other words, the new features.

#### CWGAN-GP enhanced dataset

CWGAN-GP is an improved algorithm of WGAN with conditional information input and gradient penalty terms. It generates a similar performance dataset through the generator based on the existing dataset, which is used to obscure the discriminator’s ability to distinguish between real and generated data^26^. This study combines the generated dataset with the entire original dataset to generate a new dataset, and all subsequent decomposition of load values and main model predictions by the BKA-FMD algorithm will be performed on this new dataset.

The training objective of CWGAN-GP is to minimize the Wasserstein distance, making the data distribution generated by the generator $P_{G}$ as close as possible to the real data distribution $P_{data}$. Its objective function is:

5 $$L = { mathbb{E}}_{{x \sim P_{{{\mathrm{data}}}} }} \left[ {D\left( x \right)} \right] - { mathbb{E}}_{{z \sim P_{z} }} \left[ {D\left( {G\left( z \right)} \right)} \right]$$

where ${ mathbb{E}}_{{x \sim P_{data} }} \left[ {D\left( x \right)} \right]$ is the expected score of real data on the discriminator;${ mathbb{E}}_{{z \sim P_{z} }} \left[ {D\left( {G\left( z \right)} \right)} \right]$ is the expected score of generated data on the discriminator.

To satisfy the 1-Lipschitz condition of the discriminator, CWGAN-GP incorporates a gradient penalty term into the objective function:

6 $$L_{GP} = \lambda { mathbb{E}}_{{\hat{x}}} \left[ {\left( {\begin{array}{*{20}c} {\nabla_{{\hat{x}}} D\left( {\hat{x}|y} \right)} \\ \end{array}_{2} - 1} \right)^{2} } \right]$$

where $\lambda$ is the gradient penalty coefficient; $\hat{x}$ denotes the interpolated samples randomly sampled between real and generated data; and represents the L2 norm of the gradient of the discriminator with respect to $\hat{x}$.

#### BKA-FMD load decomposition

Decomposing the target vector into multiple components for subsequent processing is a common practice. In the proposed model, the two base learners exhibit different capabilities in handling low-frequency and high-frequency datasets. Therefore, the load vector from the augmented dataset is processed using Feature Mode Decomposition (FMD), and the resulting components are grouped into high-frequency and low-frequency sets. Each group is aggregated and then separately fed into the two base learners for training, aiming to improve the accuracy of mid-term load forecasting. The hyperparameters of FMD are determined by the BKA, whose principle is detailed in Sect. BKA.

**Fig. 2:**
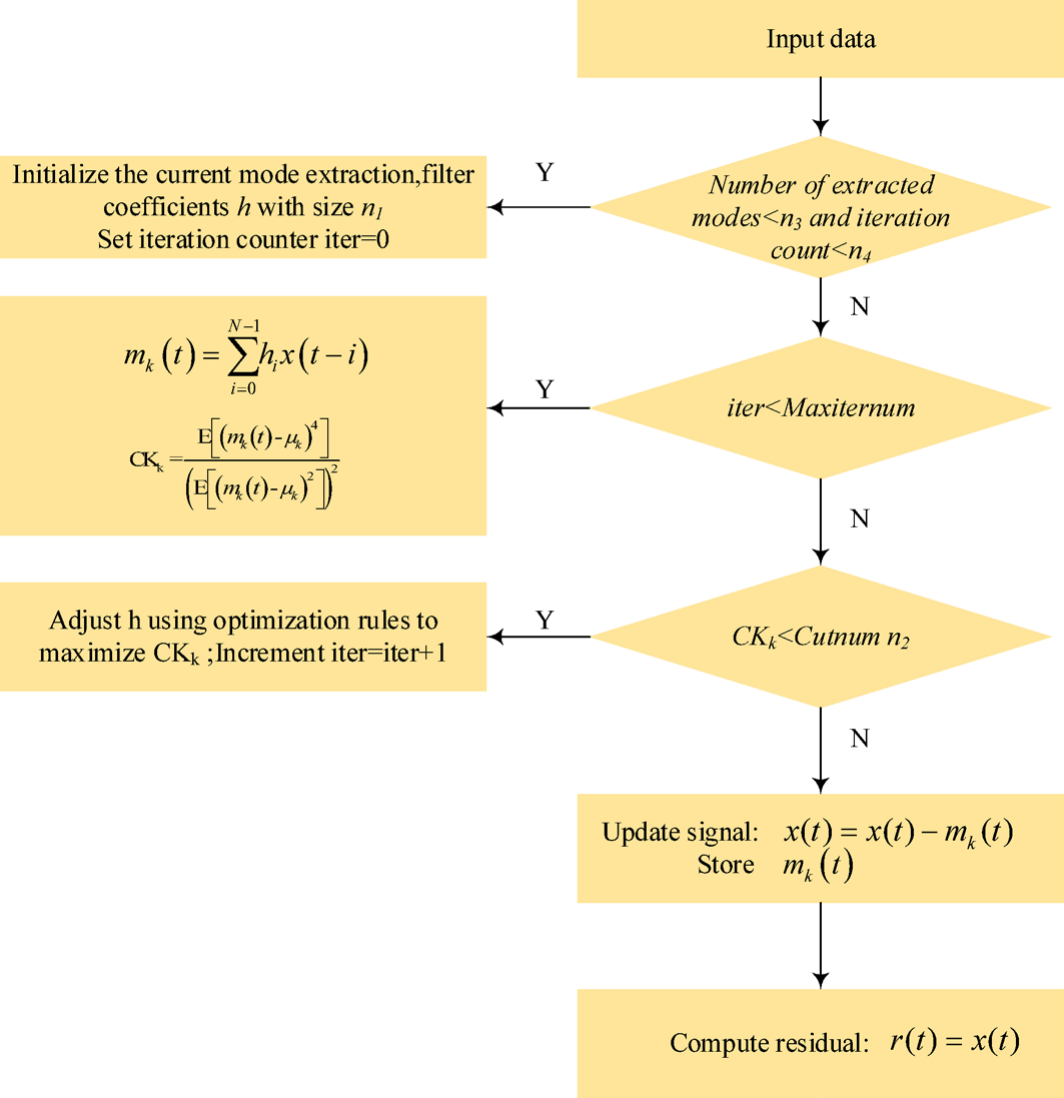
Flowchart of the FMD process.

FMD is a novel signal decomposition method that employs adaptive finite impulse response (FIR) filters to decompose signals and uses kurtosis and impulsiveness for feature extraction^27^. Compared to traditional methods like EMD and VMD, FMD performs better in handling non-stationary signals and removing mode mixing, thus providing stronger support for subsequent forecasting tasks. The flowchart of the FMD process is shown in Fig. 2. All modal components are sorted by central frequency in ascending order, with the first half forming the low-frequency part and the second half forming the high-frequency part. No fixed frequency threshold is used; the division adapts to the number of decomposed modes, matching our dual-branch architecture.

where, $h_{i}$ represents the coefficients of the filter, that is, the weights of the FIR filter. These coefficients determine the filter’s frequency response, specifying which frequency components will be extracted from the signal. $x\left( t \right)$ denotes the original signal, representing the input signal to be decomposed. $m_{k} \left( t \right)$ is the *k*-th mode, $\mu_{k}$ denotes the mean value of the mode, and ${\rm E}$ represents the expectation operator.

### Mid-term load forecasting model

#### Blending ensemble algorithm

Blending is a layered supervised learning fusion method. The key idea is to split the training set into a base model training set and a meta-model validation set^28^. First, base learners are independently trained on the training set and generate prediction results on the validation set. These prediction results are then concatenated with the original validation features to form a meta-feature matrix, which serves as the input for training the meta-model. Ultimately, the meta-model achieves a nonlinear weighted fusion of the base model outputs.

Blending isolates the data flow between base models and the meta-model, thus avoiding information leakage that may occur in cross-validation, significantly reducing the risk of overfitting. By leveraging the diversity of heterogeneous base models to capture different underlying patterns in the data and enhancing the modeling of complex nonlinear relationships through the meta-model, Blending effectively balances forecasting accuracy and robustness.

#### CNN-BiTransformer- BiLSTM

**Fig. 3:**
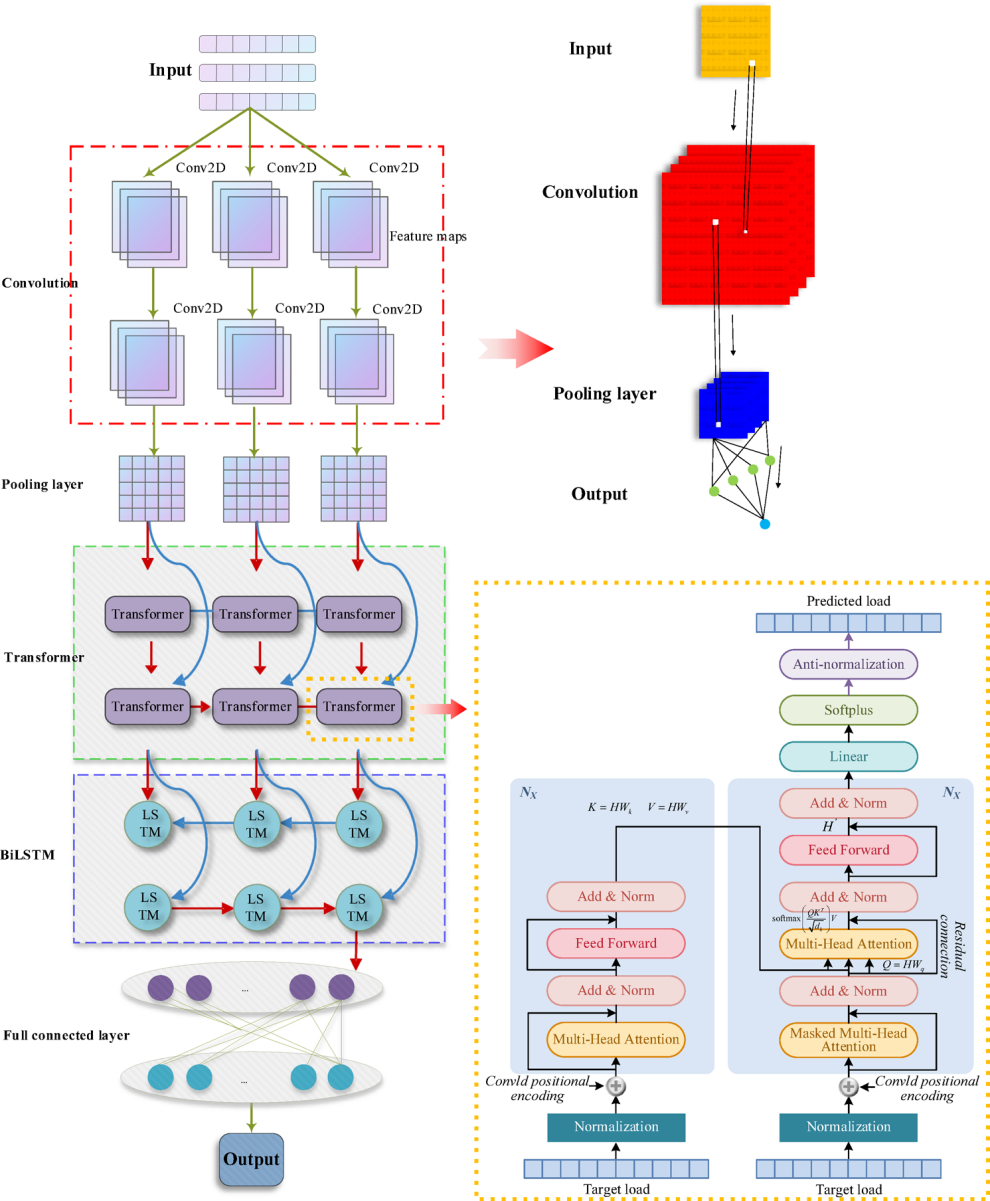
Architecture of the CNN-BiTransformer-BiLSTM Model.

CNN-BiTransformer-BiLSTM is a hybrid neural network architecture that integrates CNN, bidirectional Transformer, and bidirectional LSTM, as illustrated in Fig. 3. The CNN component extracts local patterns from the data through its local receptive field and weight-sharing mechanisms^29^. Based on the self-attention mechanism, the Transformer structure captures long-range dependencies, and its bidirectional design further enhances global information extraction^30^. The bidirectional LSTM strengthens the ability to capture complex temporal patterns through gated control and bidirectional processing^31^. The close cooperation among these three components effectively improves the forecasting accuracy for low-frequency load data^32,33^.

1. CNN

The convolution operation of the CNN in Fig. 3 is calculated as follows:

7 $$y_{t,c} = \mathop \sum \limits_{i = 0}^{k - 1} \mathop \sum \limits_{d = 1}^{D} w_{i,d,c} x_{t - i,d} + b_{c}$$

where $x_{t - i,d}$ denotes the input data at time step $t - i$ and channel $d$; $w_{i,d,c}$ represents the weight of the convolution kernel at time step $i$, input channel $d$, and output channel $c$; $b_{c}$ is the bias term for the output channel $c$; $k$ is the kernel size along the temporal dimension; $D$ is the number of input channels; $y_{t,c}$ is the extracted feature at time step $t$ for output channel $c$.

(2) BiTransformer.

The temporal modeling module of the BiTransformer is implemented as follows:

8 $$h_{t}^{BiTransformer} = \left[ {h_{t}^{{{\mathrm{forward}}}} ,h_{t}^{{{\mathrm{backward}}}} } \right]$$

where $h_{t}^{{{\mathrm{forward}}}}$ and $h_{t}^{{{\mathrm{backward}}}}$ denote the hidden states of the forward and backward Transformer units at time step $t$, respectively.

As shown in Fig. 3, each Transformer unit primarily leverages a multi-head attention mechanism to capture global temporal dependencies from local features. After linear transformations and input embeddings, the module generates the final output through residual connections, layer normalization, a position-wise feedforward network, and further residual connections and layer normalization. Specifically, $H$ represents the input sequence; $Q$, $K$, and $V$ are the query, key, and value vectors, respectively; $W_{q}$, $W_{k}$, and $W_{v}$ are the query, key, and value matrices; $d_{k}$ denotes the dimensionality of the keys; the softmax function is applied to compute the attention weights.

The output feature is denoted as $H^{\prime}$, where for the forward Transformer, it is $h_{t}^{{{\mathrm{forward}}}}$, and for the backward Transformer, it is $h_{t}^{{{\mathrm{backward}}}}$; ${\text{FF{\rm N}}}\left( \cdot \right)$ represents the processing by the feedforward neural network.

(3) BiLSTM.

BiLSTM is employed further to learn deep temporal features from the time series. LSTM is a widely used variant of recurrent neural networks, which utilizes input, forget, and output gates to control the reading, retention, and output of information. This structure effectively addresses the vanishing gradient problem and captures long-term dependencies within sequences. As illustrated in Fig. 3, BiLSTM is a bidirectional extension of the LSTM architecture. Its modular connectivity mirrors that of the BiTransformer, and its output is similarly given by:

9 $$h_{t}^{BiLSTM} = \left[ {h_{t}^{{{\mathrm{forward}}}} ,h_{t}^{{{\mathrm{backward}}}} } \right]$$

where $h_{t}^{{{\mathrm{forward}}}}$ and $h_{t}^{{{\mathrm{backward}}}}$ represent the hidden states of the forward and backward LSTM units at time step $t$, respectively.

(4) Output.

Finally, a fully connected regression layer is employed using a multi-layer neural network structure. The model is trained by minimizing the mean squared error (MSE) loss function, which is defined as:

10 $$\hat{y} = f_{FC} \left( {H^{BiLSTM} } \right) = W_{fc} H^{BiLSTM} + b_{fc}$$

where $W_{fc}$ denotes the actual value, $b_{fc}$ denotes the predicted value, and $f_{{{\mathrm{FC}}}} \left( \cdot \right)$ is the number of samples.

#### BiTCN-BiGRU-attention

**Fig. 4:**
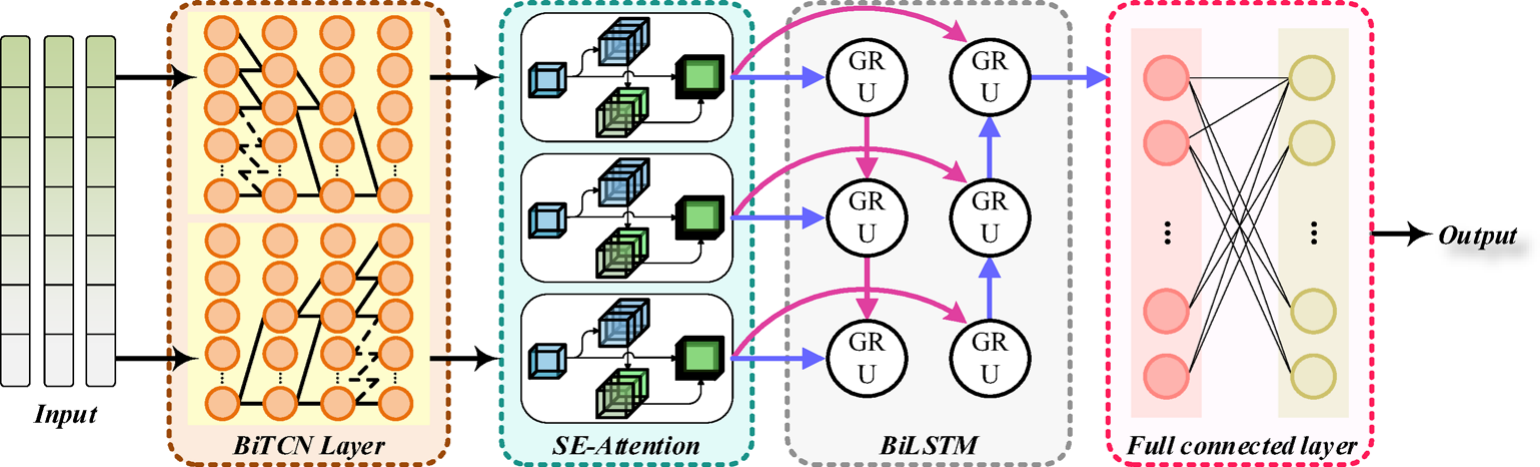
Architecture of the BiTCN-BiGRU-Attention Model.

BiTCN-BiGRU-Attention is a deep learning model that integrates a BiTCN, a BiGRU, and an attention mechanism. It is particularly effective in high-frequency load forecasting, efficiently capturing local temporal dependencies and global sequential patterns^34^. BiTCN is a convolutional architecture designed for sequential data processing^35^, capable of extracting long-term dependencies while considering past and future information. BiGRU, a bidirectional variant of the GRU, enhances the model’s ability to perceive contextual information from both directions, thereby improving prediction accuracy^36^. The attention mechanism assigns higher weights to key time steps, thereby enhancing the model’s interpretability and predictive performance^37^. The structure is illustrated in Fig. 4.

1. BiTCN.

BiTCN extends the standard Temporal Convolutional Network by deploying two parallel temporal convolutional paths. The “forward” network encodes historical observations and covariates, while the “backward” network encodes future covariates. By merging the outputs of both paths, BiTCN can capture bidirectional temporal dependencies without relying on recurrent structures. This design preserves long-range information while achieving lower parameter complexity and higher computational efficiency in sequence prediction and feature extraction tasks.

11 $$h_{t}^{BiTCN} = \left[ {h_{t}^{{{\mathrm{forward}}}} ,h_{t}^{{{\mathrm{backward}}}} } \right]$$

where $h_{t}^{{{\mathrm{forward}}}}$ and $h_{t}^{{{\mathrm{backward}}}}$ represent the hidden states of the forward and backward TCN units at time step $t$, respectively.

(2) BiGRU.

BiGRU extends the standard Gated Recurrent Unit by connecting two GRU layers in opposite directions. The forward GRU processes the input sequence chronologically, while the backward GRU processes it in reverse. The outputs from both directions are concatenated to capture past and future contextual information simultaneously. This bidirectional structure enables the model to integrate temporal dependencies from both directions at each time step, enhancing representation capability. The final temporal feature representation is obtained by either concatenating or applying a weighted average of the forward and backward hidden states:

12 $$h_{t}^{BiGRU} = \left[ {h_{t}^{{{\mathrm{forward}}}} ,h_{t}^{{{\mathrm{backward}}}} } \right]$$

where $h_{t}^{{{\mathrm{forward}}}}$ and $h_{t}^{{{\mathrm{backward}}}}$ represent the hidden states of the forward and backward GRU units at time step $t$, respectively.

(3) Attention mechanism.

The attention mechanism, inspired by the human ability to focus on salient information selectively, computes importance weights for different parts of the input, allowing the model to concentrate on the most relevant features. This enhances both feature representation and the capture of global dependencies. Specifically, query (Q), key (K), and value (V) vectors are derived through linear transformations, after which attention weights are computed. A weighted sum of the value vectors is then performed to generate the final output. This study employs the mean squared error as the regression loss function to quantify the difference between the predicted and actual values.

#### XGBoost

**Fig. 5:**
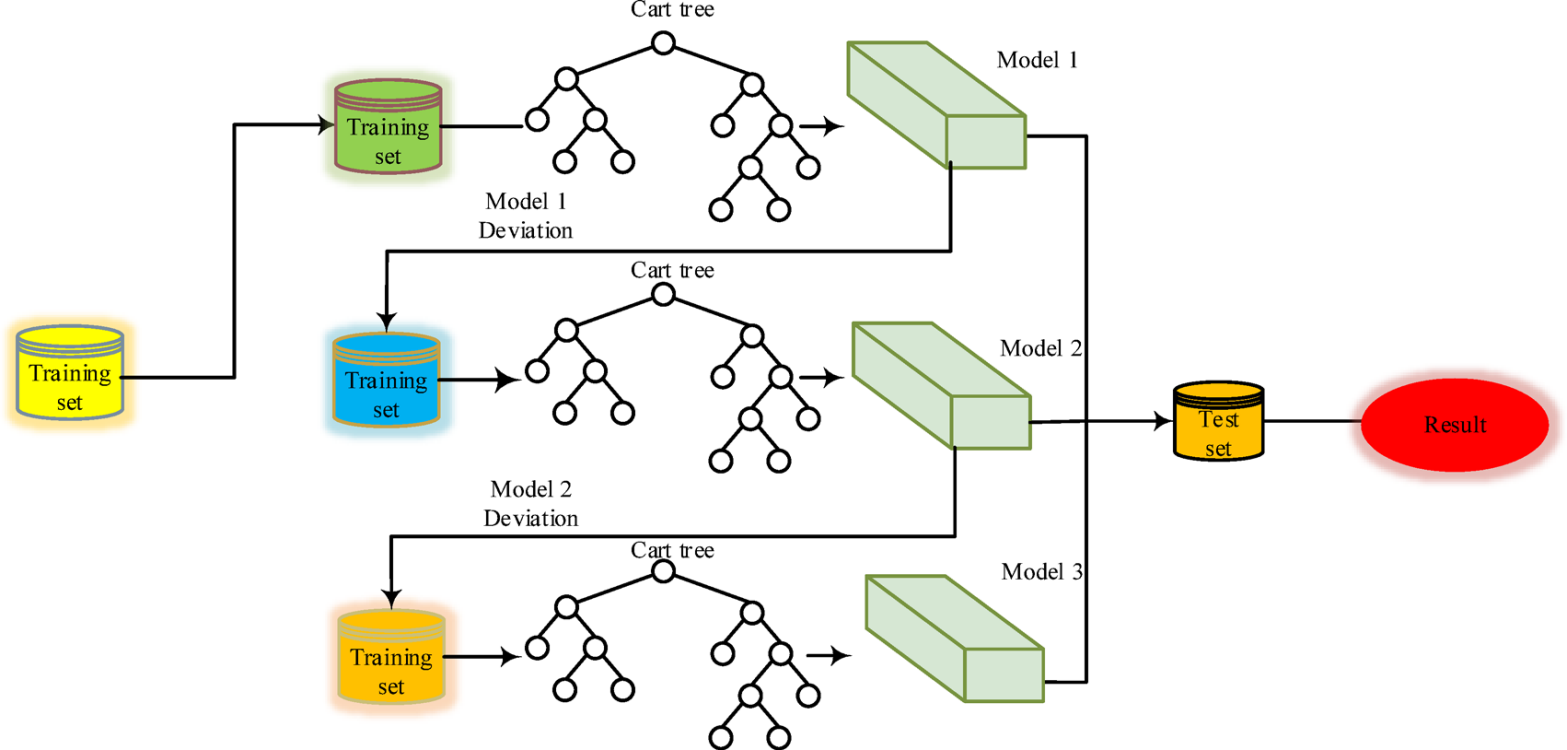
Structure of XGBoost.

XGBoost is an ensemble algorithm based on gradient boosting over decision trees, as illustrated in Fig. 5. Compared with traditional Gradient-Boosted Decision Tree algorithms, XGBoost incorporates a regularization term to control model complexity and utilizes both the first- and second-order derivatives of the loss function to accelerate convergence. These enhancements help prevent overfitting and improve prediction accuracy^38^. The final prediction of XGBoost is obtained by aggregating the outputs of all its decision trees.

#### BKA

**Fig. 6:**
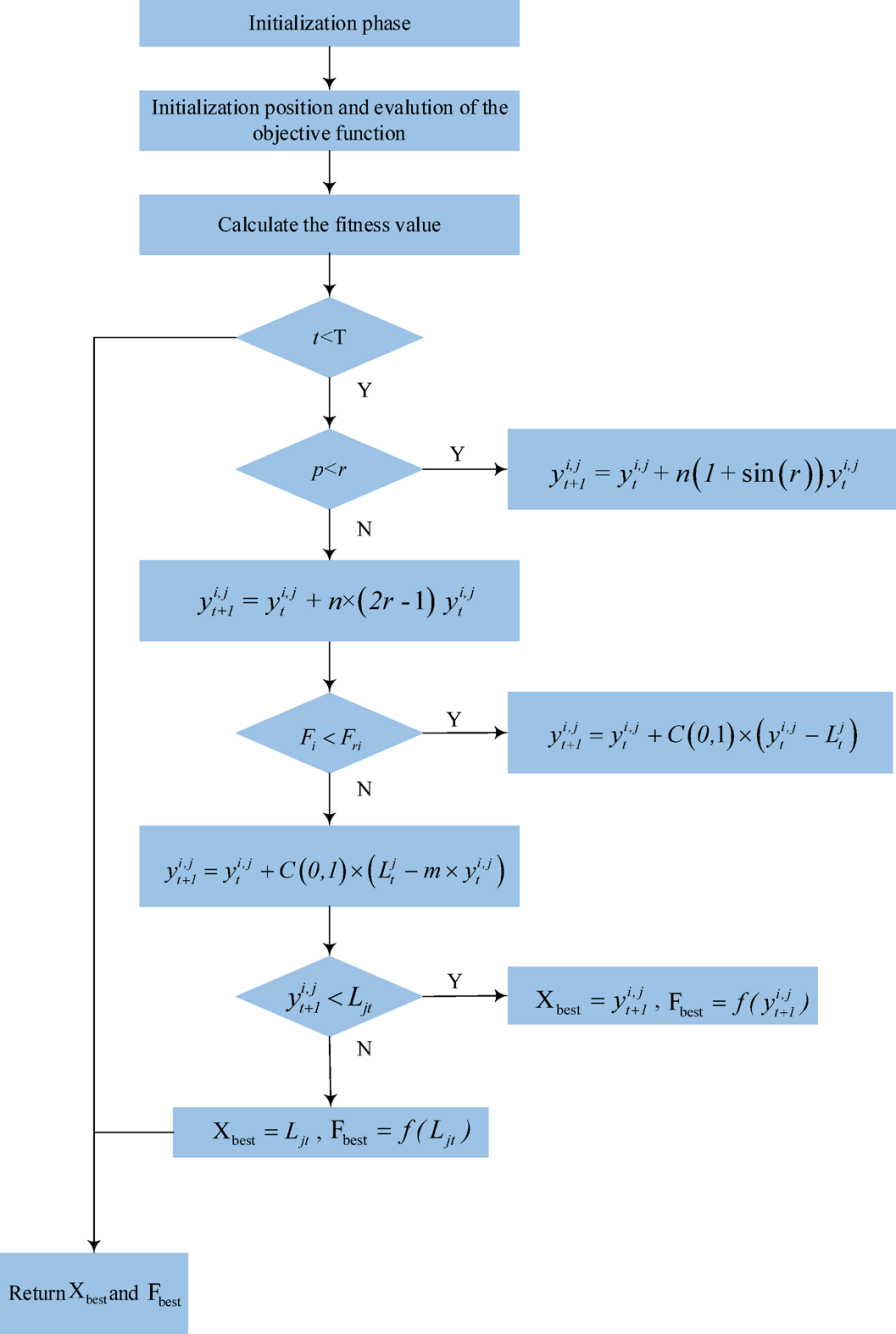
BKA flow chart.

BKA is a metaheuristic algorithm inspired by black-winged kites’ migration and predation behavior. Incorporating the Cauchy mutation strategy and leader-based guidance effectively balances global exploration and local exploitation, making it well-suited for hyperparameter optimization in other algorithms^39^. The flowchart of BKA is shown in Fig. 6.

where $X_{best}$ is the position of the global optimal solution; $F_{best}$ is the fitness value of the global optimal solution; $p$ is the attack behavior threshold that controls the switching between global exploration and local exploitation; $r$ is a uniformly generated random number; $n$ is the dynamically decaying attack behavior coefficient; $y$ is the position of the current individual, with the subscript representing the iteration number and the superscript representing the *i*-th individual and the *j*-th dimension of the individual; $F_{i}$ is the fitness value of the current individual; $F_{ri}$ is the fitness value of a random individual; $C$ is the random disturbance value generated by the standard Cauchy distribution; $m$ is the migration scaling factor; T is the maximum number of iterations.

#### Blending ensemble algorithm model based on CNN-BiTransformer-BiLSTM and BiTCN-BiGRU-attention

This study utilizes a Blending ensemble algorithm mechanism to combine the strengths of two models, CNN-BiTransformer-BiLSTM and BiTCN-BiGRU-Attention, aiming to improve time series prediction accuracy and robustness. The enhanced dataset is split into training, validation, and test sets. The test set contains half-month samples for prediction, while the remaining samples are divided between training and validation sets in a 4:1 ratio, which ensure that the validation set has enough samples for training the meta learner, resulting in stability output of the meta learner.

**Fig. 7:**
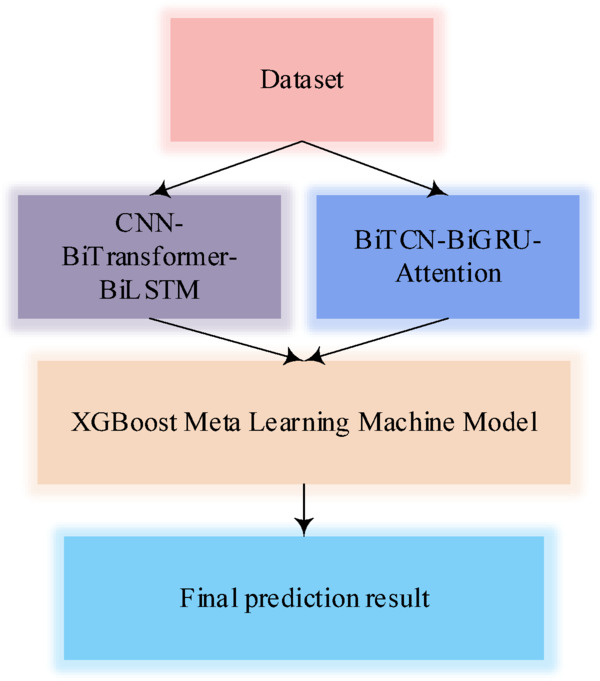
Structure of the blending ensemble algorithm model.

As shown in Fig. 7, the model training process consists of three main steps: First, CNN-BiTransformer-BiLSTM processes low-frequency load components, and BiTCN-BiGRU-Attention handles high-frequency load components in the training set, producing two prediction vectors for the validation set. Next, XGBoost is the meta-learner that trains on a new dataset created by combining these prediction vectors with the validation set. Finally, the model performs load forecasting on the test set, specifically targeting the half-month period to be predicted.

#### Computational complexity analysis

The complexity analysis of two base learners, namely CBB and BTGA, as well as the meta learner based on XGBoost, is shown in Table 2.

**Table 2:** The computational complexity of the proposed model.

Component	Time complexity	Space complexity	Operations
CBB	O(T^2^·d + T·h^2^)	48.96 KB	3.82 MFLOPs
BTGA	O(T^2^·d + T·h^2^)	31.57 KB	2.25 MFLOPs
XGBoost	O(K·D·F)	300.21 KB	700 ops

Among them, T is Input sequence length, d is Feature dimension, h is Hidden layer dimension, K is Tree count, D is Max tree depth, F is Input features. Floating point operations (FLOPs) are used for CNN and BTGA, while logical operations (ops) are used for XGBoost. Storage includes parameters and tree structures.

From Table 2, it can be seen that the time complexity of CBB accounts for a large proportion and is a key optimization focus; Meta learners require a lot of storage but have a small amount of computation; The time complexity of base learners increases exponentially with sequence length, and BTGA has lower storage and computational costs than CBB.

## Simulation experiments and analysis

### Simulation environment

The simulation experiments were conducted on the following hardware: Intel i7-13700KF CPU, NVIDIA TUF-RTX4070TiS-O16G graphics card, 32GB Kingston 3600 memory, and 2TB WD SN770 storage, with Windows 11 Version 24H2 operating system. The software used in the experiments included MATLAB R2024a (https://www.mathworks.com/products/matlab.html), Visual Studio 2022 Version 17.4 (https://visualstudio.microsoft.com/vs/), OriginPro 2024b (https://www.originlab.com/2024b), and IBM SPSS Statistics 29.0.2.0 (https://www.ibm.com/products/spss-statistics).

### Data source

This study utilized the 2023 power load dataset from a region in Belgium, combined with additional local climatic features and supplementary daily time type and weekly date type as experimental data. The time resolution was 15 min, resulting in 96 daily samples and 35,040 samples for the entire year. The dataset’s target was the actual total power load, with nine feature dimensions: humidity, maximum temperature, minimum temperature, average temperature, pressure, wind direction, wind speed, time of day, and day of week type, totaling 9 feature dimensions and 1 target dimension.

Among these, wind direction, time of day, and day of week type employed sine cyclic encoding, which is preferred over cosine-only or combined sine–cosine encoding for its natural phase alignment with feature cycle start points without extra phase correction, lower dimension with no redundancy, and simple implementation with no additional hyperparameters to ensure high reproducibility. Other features were continuous variables requiring no additional processing. Wind direction was defined with 0° representing north-to-south and positive direction counterclockwise; after cyclic encoding, the sine value of this angle was used as a feature. The time of day feature indicated the position of a sample point among the 96 daily samples, using 96 as the cycle period corresponding to a full circle for cyclic encoding. The day of week type reflected which day of the week the sample point belonged to, using the 7-day sample count (672) as the cycle period corresponding to a full circle for encoding.

This study randomly selected continuous two-week periods from each season of 2023 to serve as half-month prediction samples and act as the test set in model training. These were: Case 1: May 7–20, Case 2: July 9–22, Case 3: October 8–21, and Case 4: December 10–23.

### Data preprocessing results

**Fig. 8:**
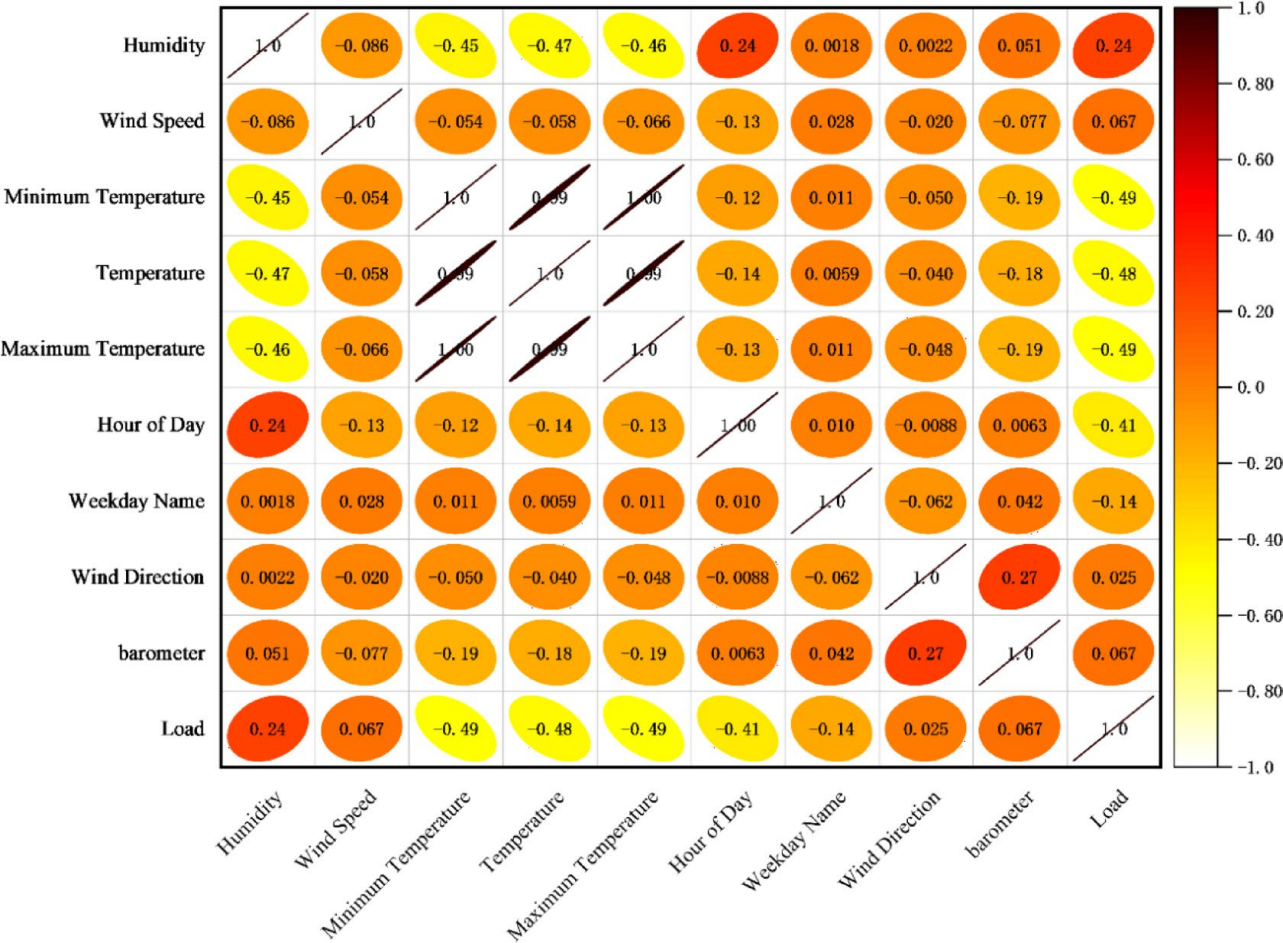
Heatmap of feature-load correlation. Drawn using OriginPro 2024b (https://www.originlab.com/2024b).

According to the principles in Section “Data Preprocessing”, outlier identification, correlation analysis, factor analysis, data augmentation, target decomposition, and normalization processing were carried out. In the first step of outlier identification, no samples with drastic changes were found, so the outlier processing step was skipped. The correlation analysis first requires a K-S test, in which the load value vector is proven to deviate significantly from the normal distribution condition. As it is a correlation analysis between various features and the target, Spearman correlation analysis is used in this paper. The visualization of the calculation results is shown in Fig. 8.

This article abandons features based on the aforementioned principles, including wind speed, wind direction, and pressure.

Next, factor analysis is performed to save computational costs, as there is a clear correlation between the highest, lowest, and average temperatures. This study conducted KMO and Bartlett’s sphericity tests, and the results are shown in Table 3. The results show that the KMO coefficient is greater than 0.7, and the Bartlett’s sphericity test is significant. Therefore, factor analysis is allowed^40^.

**Table 3:** Results of KMO and Bartlett’s test.

KMO measure of sampling adequacy	Approximate Chi-Square	Degrees of freedom	Significance
0.782	380,578.433	21	< 0.001

As shown in Table 4, this paper selects the top 5 components of the list as new features to preserve as much feature information as possible and as few features as possible. The cumulative variance explanation can reach 99.882%. Table 5 shows the linear transformation relationship between the new features and the original features.

**Table 4:** Cumulative variance explanation results.

Initial eigenvalues				Extraction sums of squared loadings			Rotation sums of squared loadings		
Component	Total	Variance percentage	Cumulative percentage	Total	Variance percentage	Cumulative percentage	Total	Variance percentage	Cumulative percentage
1	3.422	48.893	48.893	3.422	48.893	48.893	3.011	43.009	43.009
2	1.267	18.096	66.989	1.267	18.096	66.989	1.008	14.405	57.414
3	1.004	14.349	81.338	1.004	14.349	81.338	1.002	14.309	71.723
4	0.723	10.333	91.671	0.723	10.333	91.671	1.001	14.303	86.026
5	0.575	8.211	99.882	0.575	8.211	99.882	0.970	13.857	99.882
6	0.007	0.095	99.977						
7	0.002	0.023	100.000

**Table 5:** Component matrix after rotation.

Feature	Component				
	1	2	3	4	5
Min_Temperature	0.981				
Maximum_Temperature	0.979				
Temperature	0.973				
Hour_of_Day		0.991			
Wind_Direction			0.983		
Weekday_Name				0.990	
Humidity	− 0.352				0.927

**Fig. 9:**
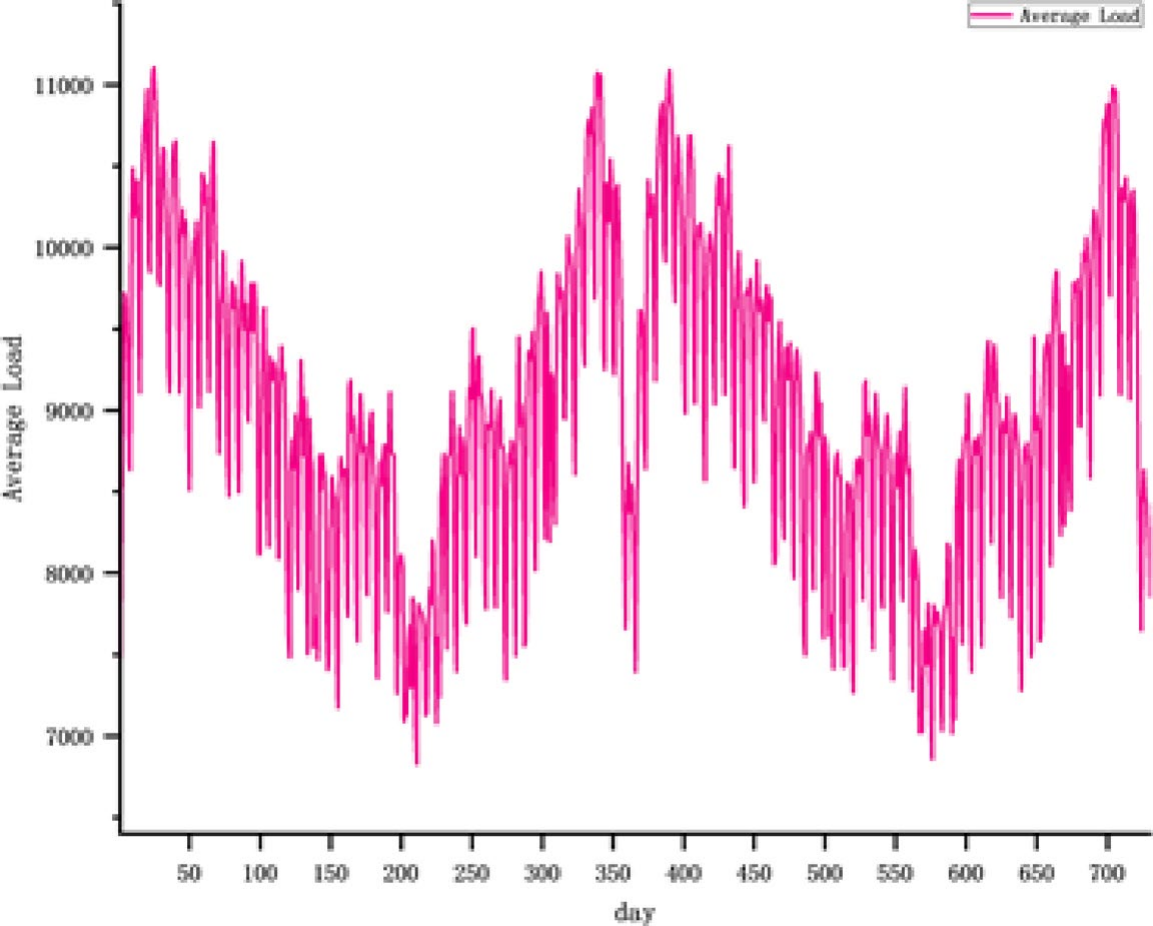
Daily average load graph of the new dataset.

While conducting factor analysis, perform data augmentation on the dataset after filtering out irrelevant features. The data generated by CWGAN-GP is concatenated in the order of the previous year’s data before the original dataset as a new dataset. The average curve of the generated new load data is shown in Fig. 9.

**Fig. 10:**
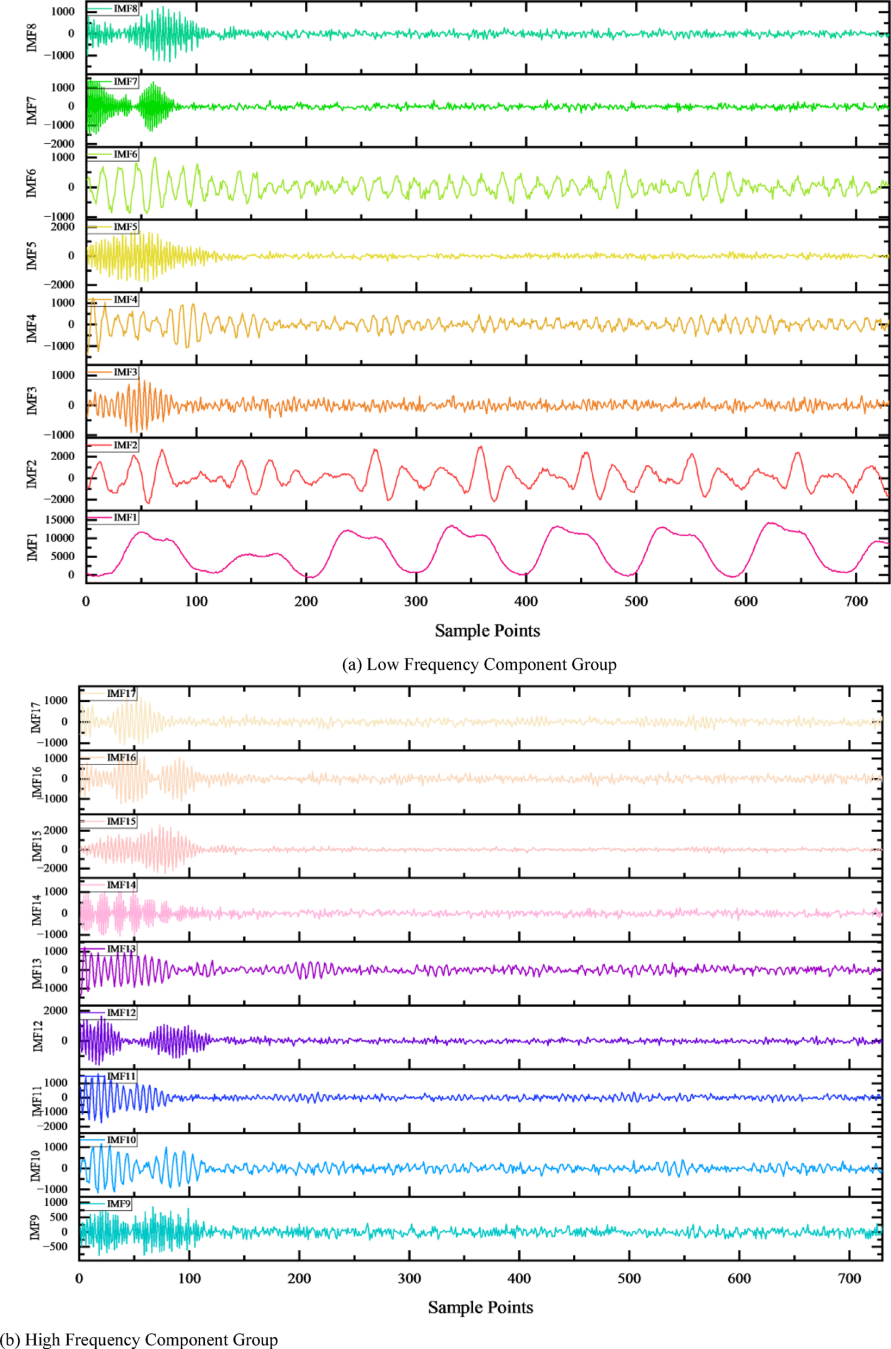
Decomposition results of BKA-FMD.

Then, the load values of the new dataset are decomposed using BKA-FMD, and the decomposed components are shown in Fig. 10. The high-frequency half components are selected to form the high-frequency component group (rounded up), and the first half components are chosen to form the low-frequency component group (rounded down), laying the foundation for importing the two base learners separately in the future. The following (b) shows the high-frequency component, and (a) shows the low-frequency component.

After preprocessing, Table 6 outlines the input formats: the first step combines features with one IMF; the second appends two output vectors from step one.

**Table 6:** Input sample structure for the first and second training steps.

	First training(CBB)	First training(BTGA)	Second training
Case 1	[37728, 6]	[37728, 6]	[9408, 8]
Case 2	[42625, 6]	[42625, 6]	[10560, 8]
Case 3	[49536, 6]	[49536, 6]	[12384, 8]
Case 4	[54432, 6]	[54432, 6]	[13536, 8]

### Optimization of BKA

**Fig. 11:**
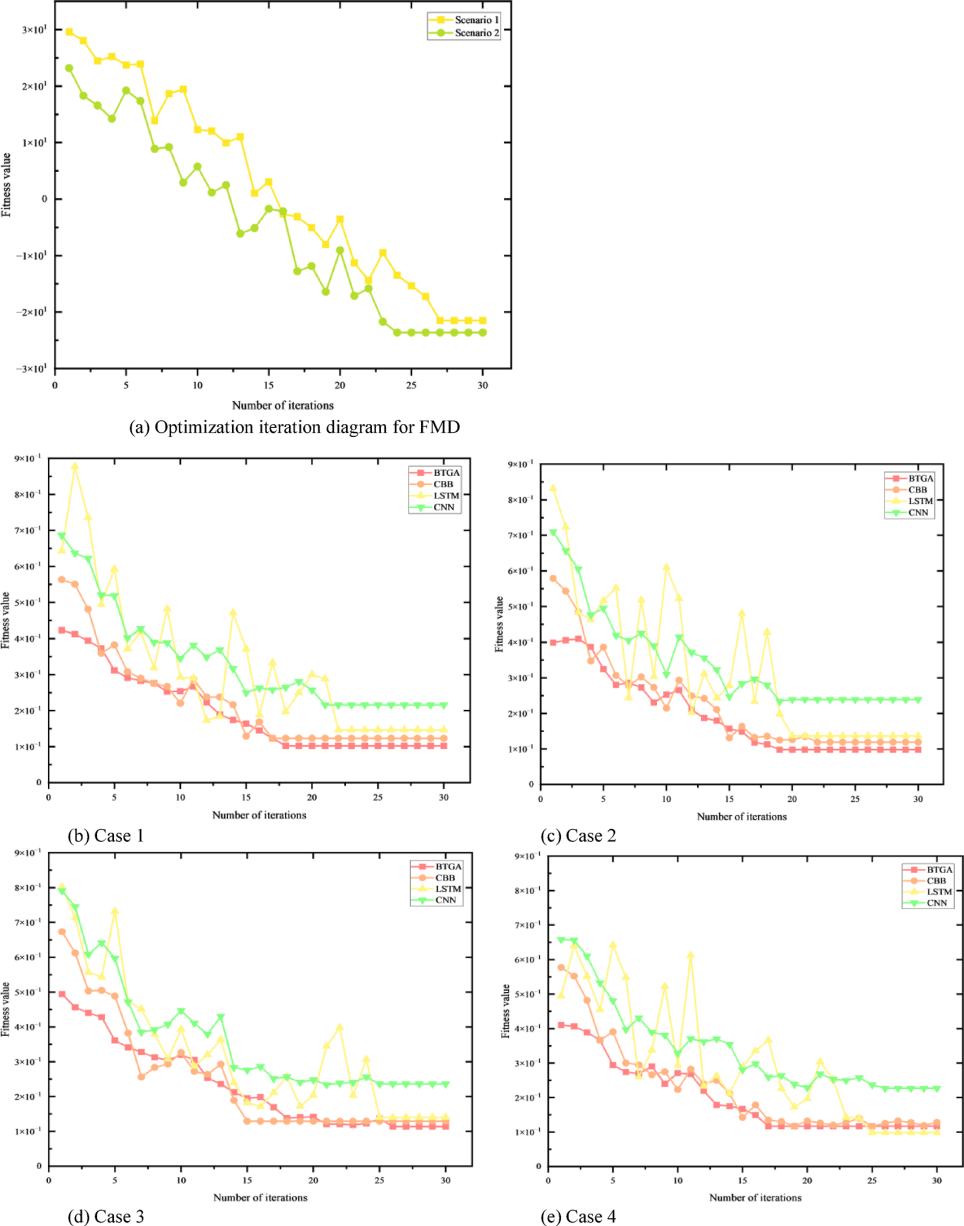
Iteration curves of BKA.

BKA is used to optimize the hyperparameters of FMD, CBB, BTGA, LSTM, and BiTCN. The latter two are for comparison algorithms, while the first three are used for the proposed model and comparison algorithms. Specifically, BKA optimizes the filter size, number of frequency bands, and number of modal components of FMD; the number of attention heads, LSTM hidden units, and initial learning rate of CBB; the number of convolution kernels, number of residual blocks, and initial learning rate of BTGA; the initial learning rate, learning rate adjustment factor, and regularization parameter of LSTM; the initial learning rate, momentum, and weight decay of BiTCN. The hyperparameter optimization iteration curves and selected results for the five algorithms are shown in Fig. 11 and Table 7. In Fig. 11a is the optimization iteration curve of FMD, where Scenario 1 and Scenario 2 represent whether the model contains WGAN; the other four, namely subFigs. b–e, show the hyperparameter iteration curves of each algorithm under the four scenarios selected in this paper. The order of hyperparameters in Table 7 is the same as stated in this paragraph.

**Table 7:** Hyperparameter selection results.

	Case 1			Case 2			Case 3			Case 4		
	Parm. 1	Parm. 2	Parm. 3	Parm. 1	Parm. 2	Parm. 3	Parm. 1	Parm. 2	Parm. 3	Parm. 1	Parm. 2	Parm. 3
FMD	188	17	18	188	17	18	188	17	18	188	17	18
BTGA	46	1	0.01874	26	3	0.0188	25	2	0.03966	29	1	0.02508
CBB	4	46	0.00364	32	74	0.0013	64	232	0.0038	1	32	0.0001
LSTM	0.0033	0.6209	0.0017	0.00738	0.8121	0.0083	0.01	1	0.0097	0.0079	0.7226	0.00169
BiTCN	0.01	0.9191	0.01	0.00787	0.9553	0.0008	0.001	0.71607	0.0027	0.0068	0.5	0.00683

### Prediction evaluation metrics

This study adopts RMSE and MAPE as evaluation metrics. Their expressions are as follows:

1. RMSE

RMSE measures the difference between predicted and actual power load values. It is susceptible to larger errors and is a commonly used metric in power load forecasting. Its expression is given as:

13 $$RMSE = \sqrt {\frac{1}{n}\mathop \sum \limits_{i = 1}^{n} \left( {\hat{y}_{i} - y_{i} } \right)^{2} }$$

where $y_{i}$ is the *i*-th actual (observed) value, $\hat{y}_{i}$ is the *i*-th predicted value, and $n$ is the total number of data points.

(2) MAPE

MAPE represents the error as a percentage and measures the model’s relative error in practical applications.

14 $$MAPE = \frac{1}{n}\mathop \sum \limits_{i = 1}^{n} \left| {\frac{{\hat{y}_{i} - y_{i} }}{{y_{i} }}} \right| \times 100{{\% }}$$

where $y_{i}$ is the *i*-th actual (observed) value, $\hat{y}_{i}$ is the *i*-th predicted value, and $n$ is the total number of data points.

### Experimental results of base learners

**Fig. 12:**
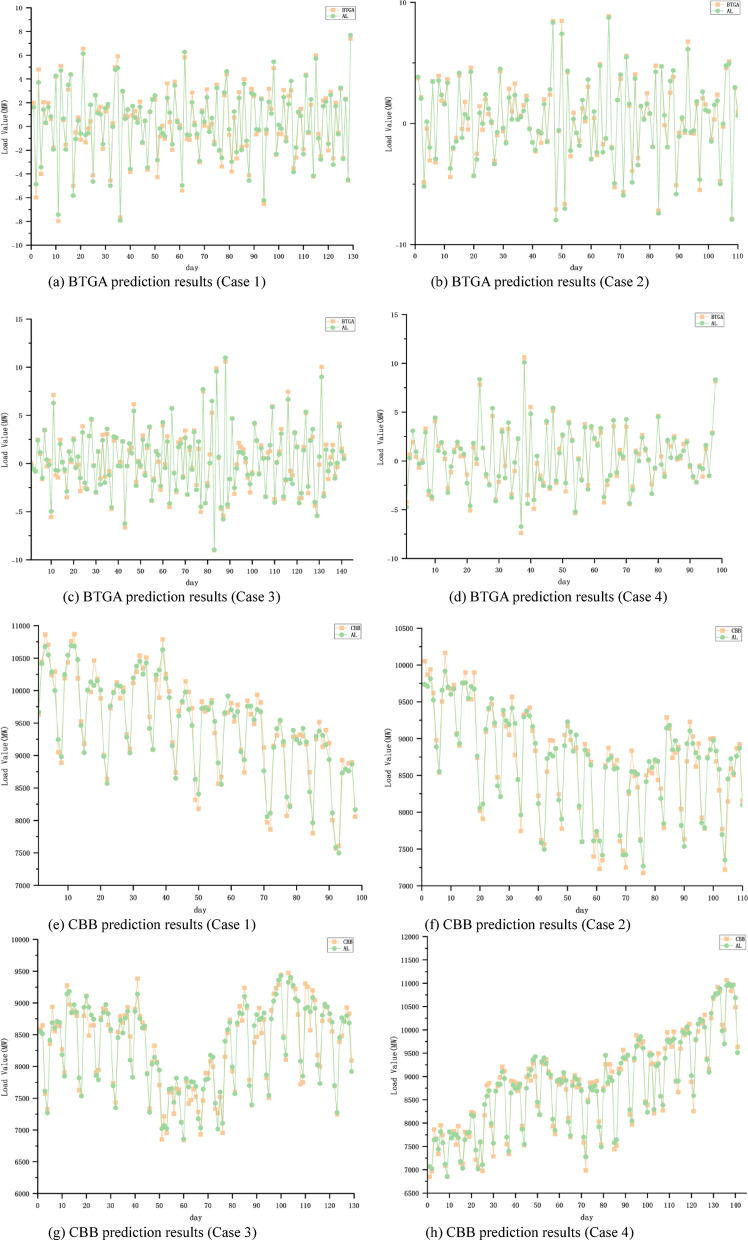
Prediction results of base learners.

CBB and BTGA process the low-frequency and high-frequency groups, respectively. The prediction values on the validation set are obtained by training on the training set, with a training-to-validation split ratio of 4:1. The test set corresponds to the period half a month before the prediction target. Based on the calculation, the number of days in the validation set for the new dataset is 98, 110, 129, and 141, respectively. In Fig. 12, subFig. a–d show the prediction performance of BTGA under four scenarios, while subFig. e–h present the prediction performance of CBB under the same four scenarios. The corresponding prediction performance results of BTGA and CBB on the validation set are listed in Table 8.

**Table 8:** Prediction results of base learners.

Scene	Case 1		Case 2		Case 3		Case 4	
	BTGA	CBB	BTGA	CBB	BTGA	CBB	BTGA	CBB
MAPE	0.117	0.207	0.126	0.216	0.119	0.192	0.104	0.175
RMSE	1206.42	2157.66	1247.62	2136.21	1251.86	2046.94	1242.63	2139.25

### Ablation study of the overall model

To verify the accuracy and necessity of each component of the proposed model, two sets of ablation experiments were designed, namely Group A and Group B. Group A includes the full version of the model proposed in this paper and models with one component missing or altered; Group B includes the full model and experiments with two components missing or altered. For models containing BKA, the hyperparameters of each component are selected according to Sect. Optimization of BKA. For those not containing BKA, the hyperparameters are randomly selected near the values optimized by BKA.

1. Ablation experiment group A.

From the curve of the actual values, it can be seen that the overall load exhibits an apparent periodic rise and fall with a period of 7 days, which is caused by the different socio-economic activities between weekdays and weekends. From the different forecast half-months, it can be observed that the winter load is significantly higher than that of other months. The possible reason is that Belgium is located in Western Europe, which has a temperate maritime climate, and most indoor spaces are heated in winter, leading to a specific range of load increase. In contrast, the summer temperature is mild, and the use of air conditioners is not widespread. For the full names of each algorithm component, please refer to the abbreviation table.

**Fig. 13:**
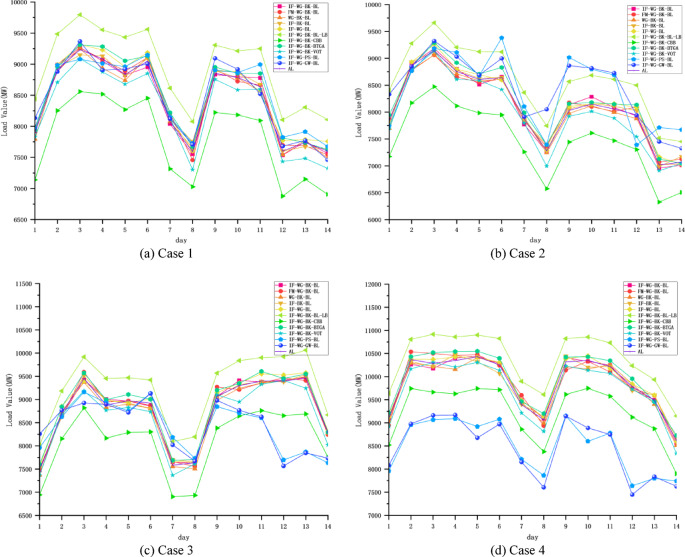
Results of ablation experiments in group A.

Figure 13 and Table 9 show the prediction results of Group A. In Group A experiments, the model proposed in this paper is IF-WG-BK-BL. Its RMSE shows a trend of first increasing and then gradually decreasing, while the MAPE shows a trend of first slowly increasing and then continuously decreasing. This indicates that with the increase of load samples, the model initially shows slight overfitting but gradually identifies new variation patterns. It forms a validation group with FM-WG-BK-BL to verify that BKA-optimized FMD can improve prediction accuracy. IF-WG-BK-BL and FM-WG-BK-BL are compared with WG-BK-BL to verify that both FMD and IFMD improve model performance; Compared with IF-BK-BL, it verifies that enhancing the dataset with CWGAN-GP indeed improves the generalization ability of the model; Compared with IF-WG-BL, it verifies that after optimizing the hyperparameters of each component using the BKA algorithm, the model’s convergence accuracy improves; The difference from IF-WG-BK-BL-LB is that the two base learners are replaced with LSTM and BiTCN, verifying the necessity of using CNN-BiTransformer-BiLSTM and BiTCN-BiGRU-Attention as the two base learners under the Blending ensemble model. The differences with IF-WG-BK-CBB and IF-WG-BK-BTGA are that the main forecasting model, the Blending ensemble algorithm, and its two base learners are entirely replaced by CNN-BiTransformer-BiLSTM and BiTCN-BiGRU-Attention, respectively, to verify that the ensemble algorithm has better accuracy than using a single base learner alone; Compared with IF-WG-BK-VOT, where the two base learners remain unchanged and only the ensemble model type is replaced by Voting, it verifies that in mid-term load forecasting, Blending has a better ability to converge to the possible true values than the simpler Voting ensemble method; The differences with IF-WG-PS-BL and IF-WG-GW-BL lie in using basic PSO and GWO algorithms for selecting hyperparameters of each component, validating that the BKA algorithm is more effective in this paper’s mid-term load forecasting model.

**Table 9:** Performance metrics of ablation experiments in group A.

Scene	Evaluation Index	IF-WG-BK-BL	FM-WG-BK-BL	WG-BK-BL	IF-BK-BL	IF-WG-BL	IF-WG-BK-BL-LB	IF-WG-BK-CBB	IF-WG-BK-BTGA	IF-WG-BK-VOT	IF-WG-PS-BL	IF-WG-GW-BL
Case 1	RMSE	538.6936	978.8023	702.5692	723.2087	652.216	800.1736	926.844	752.188	856.7256	1613.025	1884.816
	MAPE	0.05218	0.094297	0.067189	0.070625	0.062528	0.078821	0.091546	0.072034	0.082261	0.156097	0.181243
Case 2	RMSE	557.8848	1009.539	700.0991	715.5499	658.8288	807.1642	919.4397	743.6298	860.584	1914.96	2015.755
	MAPE	0.056135	0.100309	0.069592	0.070632	0.065988	0.082117	0.095296	0.073407	0.08582	0.192079	0.199974
Case 3	RMSE	551.4785	978.3191	712.9557	742.3158	681.2218	812.5858	934.1827	748.0053	878.2541	1781.726	2017.026
	MAPE	0.051316	0.090716	0.066565	0.067664	0.064125	0.077319	0.089265	0.068545	0.082339	0.165093	0.184887
Case 4	RMSE	554.8088	1013.994	682.6447	725.5848	648.337	794.4493	926.9184	769.4872	849.5519	2187.443	2383.162
	MAPE	0.045601	0.084304	0.055639	0.059274	0.053342	0.067684	0.078375	0.06311	0.070579	0.180212	0.198876

(2) Ablation experiment group B.

**Fig. 14:**
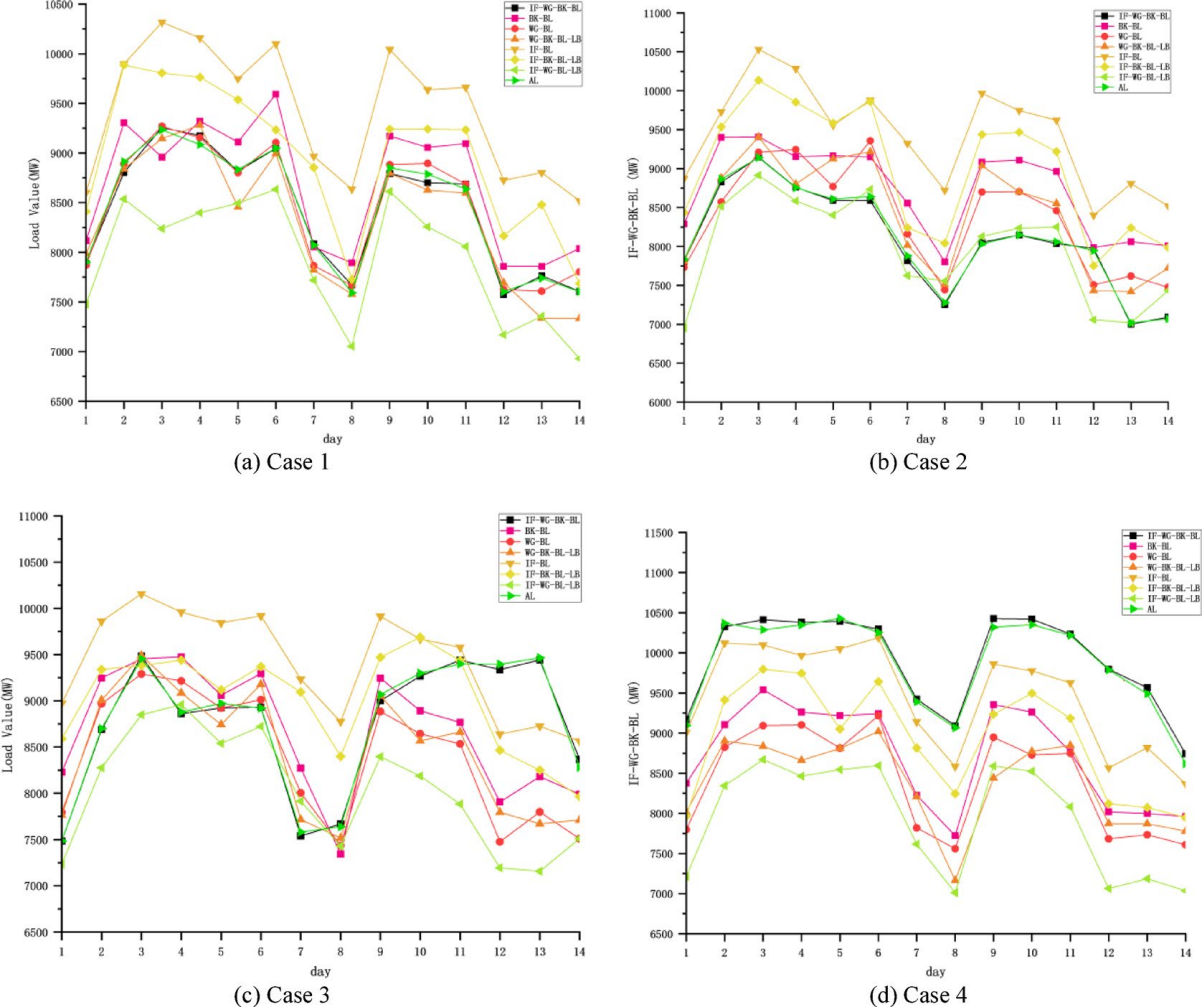
Results of ablation experiments in group B.

The prediction results of Group B are shown in Fig. 14 and Table 10. The Group B experiments refer to ablation experiment sets where two different components of the proposed model are removed or modified. For each model, if BKA is included, the component hyperparameters are selected according to Section “Optimization of BKA”; if BKA is not included, the hyperparameters are randomly selected near those obtained by BKA.

**Table 10:** Performance metrics of ablation experiments in group B.

Scene	Evaluation Index	IF-WG-BK-BL	BK-BL	WG-BL	WG-BK-BL-LB	IF-BL	IF-BK-BL-LB	IF-WG-BL-LB
Case 1	RMSE	547.43	1528.748	1260.173	2018.493	1611.779	2442.823	2498.905
	MAPE	0.0523	0.145084	0.121246	0.197772	0.158252	0.231377	0.244189
Case 2	RMSE	551.60	1722.042	1357.551	2108.356	1932.524	2619.962	2580.556
	MAPE	0.0559	0.173098	0.135058	0.210265	0.200632	0.263166	0.256509
Case 3	RMSE	542.87	1706.046	1571.339	2216.586	1715.72	2485.45	2839.754
	MAPE	0.0522	0.15642	0.144208	0.205798	0.162125	0.230156	0.260093
Case 4	RMSE	559.80	1961.93	2015.686	2600.698	1530.474	2650.713	3225.628
	MAPE	0.0460	0.158149	0.168796	0.21301	0.124141	0.216513	0.266516

As can be seen from the figure, all models generally capture the trend pattern of the actual load variation to some extent across the four scenarios. However, regarding numerical accuracy, the proposed model, WG-BL, and WG-BK-BL are more closely aligned with the actual values. In contrast, IF-BL, IF-BK-BL, IF-WG-BL, and BK-BL show larger deviations from the actual values. Among them, the poorer performance of IF-WG-BL compared to WG-BL may be because the hyperparameters of the two base learners were not obtained through an optimization algorithm, and the decomposition by IFMD amplified this error.

### Comparison experiments of the overall model

**Fig. 15:**
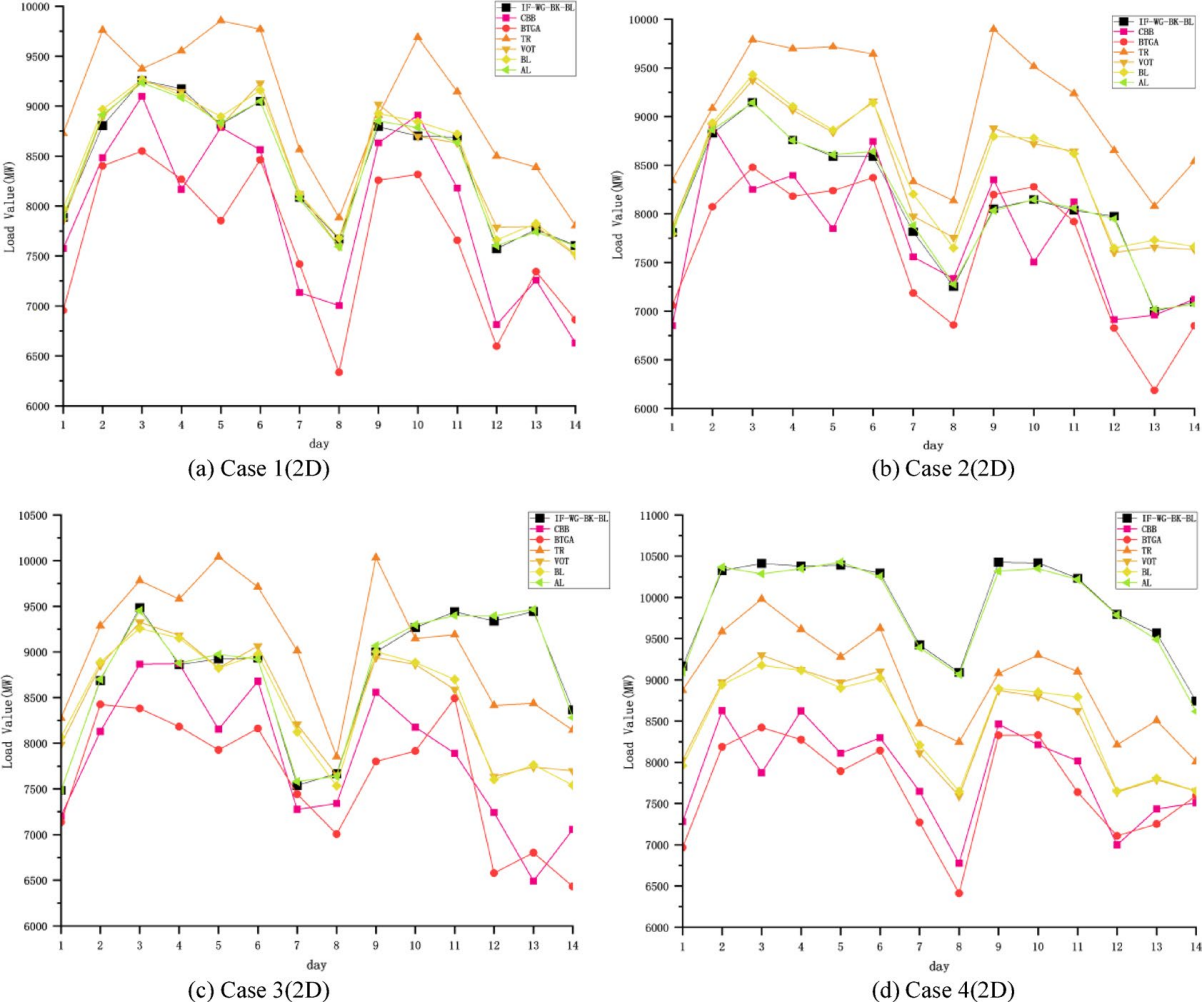
Results of comparison experiment.

In this study, several typical and advanced algorithms from recent years were selected for comparison, with the results shown in Fig. 15 and Table 11. As seen in the figure, the Blending ensemble algorithm (using LSTM and BiTCN as base learners) and the Voting ensemble algorithm (with the same base learners as the model in this paper) show more minor prediction errors for the actual load in Case 1 and Case 2 compared to the proposed algorithm. This is because ensemble algorithms generally achieve higher prediction accuracy than individual models. However, in Case 3 and Case 4, all models except those proposed in this paper show significant deviations. This is because other models have fewer datasets and simpler structures, which cannot capture more complex patterns, resulting in overfitting.

**Table 11:** Performance metrics of comparison experiment.

Scene	Evaluation Index	IF-WG-BK-BL	CBB	BTGA	TR	VOT	BL-LB
Case 1	RMSE	547.43	2988.435	2457.73	2375.705	743.5607	750.5058
	MAPE	0.0523	0.284675	0.234427	0.227267	0.071931	0.071652
Case 2	RMSE	551.60	3108.467	2375.51	2513.816	947.5794	956.3734
	MAPE	0.0559	0.312952	0.235988	0.248947	0.094213	0.09625
Case 3	RMSE	542.87	3314.987	2683.276	2503.359	1164.036	1166.601
	MAPE	0.0522	0.309704	0.247844	0.232664	0.103854	0.103664
Case 4	RMSE	559.80	3711.011	3260.263	2547.103	1690.768	1692.427
	MAPE	0.0460	0.31035	0.27312	0.2099	0.143494	0.143995

## Discussion

Previous studies have developed various ensemble and optimization techniques to improve load forecasting performance. For example, Dostmohammadi et al. ^12^ adopted a GA-stacking ensemble of diverse learners for energy consumption forecasting, and Ren et al. ^13^ proposed a stacking ensemble framework with deep reinforcement learning for multi-energy load forecasting, both of which achieved effective accuracy improvement in their respective scenarios. Compared with these existing methods, our model innovatively integrates FMD decomposition for frequency-domain feature extraction, CWGAN-GP for conditional data augmentation, and BKA for adaptive hyperparameter optimization, and the dual-branch CBB and BTGA architecture effectively captures low- and high-frequency load components respectively. Experimental results show that our model reduces RMSE by at least 3.02% and MAPE by at least 17.7% against mainstream comparison algorithms, which proves that the proposed integrated hybrid model has higher accuracy and stronger generalization ability for mid-term load forecasting than single models and traditional ensemble methods.

However, the model constructed in this paper does not take data privacy protection into account during model training and application, which may lead to potential data leakage issues in practical power grid scenarios with sensitive user and operation data.

In future work, the proposed model can be further extended in several directions closely related to load forecasting. Firstly, introducing additional exogenous variables such as economic indicators, renewable energy generation data, and consumer behavior factors can enhance the model’s ability to capture complex demand dynamics. Secondly, transfer learning and domain adaptation techniques can be used to improve the generalization ability of the model in different regions or seasons. Thirdly, a real-time adaptive prediction framework can be developed to handle non-stationary and rapidly changing load patterns. In addition, from the perspective of data security, future research can explore integrating differential privacy, federated learning, and homomorphic encryption into the training process to achieve privacy preserving predictions while maintaining prediction accuracy.

## Conclusion

This paper focuses on medium-term load forecasting and proposes a Blending ensemble load forecasting model based on BKA and CWGAN-GP, using CNN-BiTransformer-BiLSTM and BiTCN-BiGRU-Attention as base learners. Based on the simulation results, the conclusions can be summarized as follows:

1. The model containing the BKA optimization algorithm achieves higher prediction accuracy than models without optimization or those using basic PSO and GWO algorithms.
2. IFMD can improve prediction accuracy under suitable hyperparameters; however, it may reduce accuracy if hyperparameters are improperly selected.
3. Using CWGAN-GP improves data generalization ability. Compared to IF-BK-BL without CWGAN-GP, RMSE is reduced by 24.21%, and MAPE is reduced by 23.48%.
4. Blending ensemble leverages the advantages of both base learners, and its base models, CBB and BTGA, cannot be easily replaced by simpler models.
5. The proposed model has higher accuracy, robustness, and generalization ability. RMSE can be reduced by at least 3.02% in the three experimental groups, and MAPE can be reduced by at least 17.7%.

To sum up, the proposed integrated deep learning model effectively addresses the core challenges of mid-term load forecasting under load volatility and limited historical data scenarios, and provides a solid theoretical basis support for the optimal dispatching, economic operation and planning construction of the new power system.

## Data Availability

The analysis result data used to support the findings of this study are included within the article.

## Abbreviations

2D: Two-Dimensional diagram
3D: Three-Dimensional diagram
AutoML: Automated machine learning
AL: Actual load value
ANN: Artificial neural network
BKA(BK): Black-Winged Kite Algorithm
BiGRU: Bidirectional gated recurrent unit
BiLSTM: Bidirectional long short-term memory
BiTCN: Bidirectional temporal convolutional network
BL: Blending ensemble algorithm based on CBB and BTGA as base learners
BL-LB: Blending ensemble algorithm based on LSTM and BiTCN as base learners
BTGA: BiTCN-BiGRU-attention
CNN: Convolutional neural network
CBB: CNN-BiTransformer-BiLSTM
CEEMDAN: Complete ensemble empirical mode decomposition with adaptive noise
CGAN: Conditional generative adversarial network
CWGAN-GP(WG): Conditional Wasserstein generative adversarial network with gradient penalty
DDPG: Deep deterministic policy gradient
DT: Decision tree
EMD: Empirical mode decomposition
EEMD: Ensemble empirical mode decomposition
EWT: Empirical wavelet transform
FMD(FM): Feature mode decomposition
GAN: Wasserstein generative adversarial
GBR: Gradient boosting regression
GBT: Gradient boosting tree
GWO(GW): Grey wolf optimizer
IFMD(IF): BKA improved feature mode decomposition
IMFs: Intrinsic mode functions
IMPA: Iterative model path algorithm
KFCM: Kernel fuzzy C-means
K-S test: Kolmogorov–Smirnov test
KMO test: Kaiser–Meyer–Olkin test
KNN: K-nearest neighbors
MAPE: Mean absolute percentage error
ODE: Ordinary differential equation
PGBM: Proximal gradient boosting machine
PSO(PS): Particle swarm optimization
RF: Random forest
RMSE: Root mean squared error
STL: Seasonal and trend decomposition using Loess
VOT: Voting ensemble algorithm based on CBB and BTGA as base learners
XGBoost: EXtreme gradient boosting
